# Nuclear Receptor HNF4α Binding Sequences are Widespread in Alu Repeats

**DOI:** 10.1186/1471-2164-12-560

**Published:** 2011-11-15

**Authors:** Eugene Bolotin, Karthikeyani Chellappa, Wendy Hwang-Verslues, Jake M Schnabl, Chuhu Yang, Frances M Sladek

**Affiliations:** 1 Department of Cell Biology and Neuroscience, University of California, Riverside, Riverside, CA, 92521, USA; 2 Institute for Integrative Genome Biology, University of California, Riverside, Riverside, CA, 92521, USA; 3 CHORI (Children's Hospital Oakland Research Institute), 5700 Martin Luther King Jr Way, Oakland, CA 94609, USA; 4 Genomics Research Center, Academia Sinica, No. 128 Academia Road, Section 2, Nankang District, Taipei 115, Taiwan; 5 Roche Diagnostics Shanghai Limited, 1045 Central Huaihai Road, Shanghai 200031, China

## Abstract

**Background:**

Alu repeats, which account for ~10% of the human genome, were originally considered to be junk DNA. Recent studies, however, suggest that they may contain transcription factor binding sites and hence possibly play a role in regulating gene expression.

**Results:**

Here, we show that binding sites for a highly conserved member of the nuclear receptor superfamily of ligand-dependent transcription factors, hepatocyte nuclear factor 4alpha (HNF4α, NR2A1), are highly prevalent in Alu repeats. We employ high throughput protein binding microarrays (PBMs) to show that HNF4α binds > 66 unique sequences in Alu repeats that are present in ~1.2 million locations in the human genome. We use chromatin immunoprecipitation (ChIP) to demonstrate that HNF4α binds Alu elements in the promoters of target genes (*ABCC3, APOA4, APOM, ATPIF1, CANX, FEMT1A, GSTM4, IL32, IP6K2, PRLR, PRODH2, SOCS2, TTR*) and luciferase assays to show that at least some of those Alu elements can modulate HNF4α-mediated transactivation *in vivo *(*APOM, PRODH2, TTR, APOA4*). HNF4α-Alu elements are enriched in promoters of genes involved in RNA processing and a sizeable fraction are in regions of accessible chromatin. Comparative genomics analysis suggests that there may have been a gain in HNF4α binding sites in Alu elements during evolution and that non Alu repeats, such as Tiggers, also contain HNF4α sites.

**Conclusions:**

Our findings suggest that HNF4α, in addition to regulating gene expression via high affinity binding sites, may also modulate transcription via low affinity sites in Alu repeats.

## Background

As much as 50% of the ~3 billion base pairs in the human genome may be derived from repetitive DNA sequence [1]. While repetitive DNA is often referred to as "junk" DNA, even when that term was originally coined it was hypothesized that junk DNA may play an active role in genome function [2]. The notion that repetitive DNA may play a regulatory role and be involved in the evolution of gene regulation was also postulated early on, although it was not until recently that there was evidence to support those ideas [3-5].

A major category of repetitive DNA is short interspersed nuclear elements (SINEs), which are believed to have originated from the 7SL RNA gene that is part of the ribosome complex [6]. In the human genome, the largest class of SINEs are Alu repeats, which at ~1.2 million copies account for ~10% of the human genome [1]. Alu elements were first characterized as ~300 nucleotide repetitive sequences that contain an AluI restriction site (5'-AGCT-3') from the bacterium *Arthrobacter luteus *[7,8]. Alu elements, which are still mobile in the human genome by virtue of the action of a LINE-1 reverse transcriptase [9], are a relatively recent occurrence evolutionarily. They are found exclusively in primates, including humans, and hence are postulated to have entered the mammalian genome ~60-65 million years ago [10].

Alu elements have been implicated in several human diseases including leukemia, hemophilia and breast cancer, suggesting that their impact on human health may be significant [11]. There are several well characterized examples of Alu insertions affecting splicing patterns and hence protein function [12]. A variety of transcription factor (TF) binding sites (TFBSs) have also been characterized in Alu elements, including sites for YY1 [13], Sp1 [14], tumor suppressor p53 [15], homeodomain and TATA binding proteins [16]. Nuclear receptors (NR), which belong to a superfamily of ligand-dependent TFs, have also been found to have binding sites in Alu elements: retinoid acid receptor (RAR, NR1B) [17], estrogen receptor (ER, NR3A) [18,19], progesterone receptor (PR, NR3C3) [20] and vitamin D receptor (VDR, NR1I1) [21]. Alu insertions have also been shown to alter the expression of at least six human genes: CD8a (*CD8A)*, keratin 18 (*KRT18)*, parathyroid hormone (*PTH)*, Wilm's tumor 1 (*WT1)*, receptor for Fc fragment of IgE, high affinity I, gamma polypeptide *(FCER1G) *and breast cancer 1, early onset (*BRCA1) *[22]. Therefore, Alu sequences may regulate the level of transcripts and hence proteins in the cell, as well as the function of those proteins.

Hepatocyte nuclear factor 4 alpha, (HNF4α, NR2A1) is a member of the NR superfamily that is highly expressed in the liver, as well as the kidney, intestine (large and small), pancreas and stomach [23]. HNF4α is best known for its role in the adult liver and pancreas, as well as in early development [24,25]; it also has an emerging role in the gut [26-28]. The *HNF4Α *gene is mutated in an inherited form of type 2 diabetes, maturity onset diabetes of the young 1 (MODY1) [29], and was recently identified as a susceptibility locus in inflammatory bowel disease (IBD) [30]. Mutations in HNF4α binding sites have also been directly linked to human diseases, including hemophilia and MODY3 [31,32]. Many NRs are common drug targets [33]; the recent identification of the endogenous ligand of HNF4α that binds in a reversible fashion also makes HNF4α a potential drug target [34,35].

In addition to its medical relevance, HNF4α also appears to play a unique role in the evolution of NRs. It is highly conserved across species, with 100% amino acid conservation in the DNA binding domain of all mammalian HNF4α. While HNF4α is most similar to the retinoid × receptor alpha (RXRα, NR2B1), unlike many other NRs, it does not heterodimerize with RXR. Rather, it binds DNA in the form of direct repeats separated by one nucleotide (DR1, AGGTCAxAGGTCA) exclusively as a homodimer [36]. HNF4α has been found in every animal organism examined thus far, including sponge and coral [37], and has been postulated to be the ancestor of the entire NR family [38].

Many hundreds of HNF4α target genes have been identified by both classical promoter analysis as well as more modern genome-wide studies [32,39-41]. During one such genomic study, we observed a very uneven frequency profile of individual HNF4α binding sequences [42]. Specifically, we noted that a certain DNA sequence designated H4.141 (5'-AGGCTGaAGTGCA-3') was > 100-fold overrepresented compared to other HNF4α binding sites in the human, but not the mouse, genome (see additional file 1: **Figure S1**). In the current study, we investigate the notion that these and other HNF4α binding sequences are in Alu repeats. We use the powerful high throughput technology of protein binding micorarrays (PBMs) to show that HNF4α does indeed bind numerous sequences in Alu repeats *in vitro*. We perform ChIP and luciferase assays to show that HNF4α binds at least some Alu sequences *in vivo *and that those binding events are associated with transcriptional activation. Finally, we investigate accessibility of these sites by correlation with DNase hypsersensitivity data and evolutionary conservation by comparative genomic analysis.

## Results

### HNF4α binds Alu repeats *in vitro*

**Figure 1:**
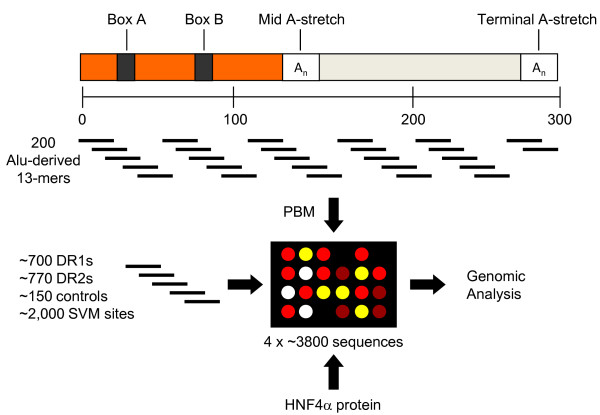
**Schematic diagram of the protein binding microarray (PBM) designed to test the ability of HNF4α to bind Alu-derived DNA sequences**. Top, schematic structure of a generic Alu element (~300 nt long) comprised of two related, but non identical monomers, the right and left arms (adapted from [75]). Box A and B are RNA Pol III internal promoters. Relative positions of the 200 Alu-derived 13-mers incorporated into PBM3 are also shown. Bottom, remaining DNA probes on PBM3 and workflow.

Since genome-wide location analysis (i.e., ChIP-chip/seq) often filters out or cannot distinguish the exact location of TF binding events in highly repetitive DNA, we took a combined *in vitro/in silico *approach to determine whether HNF4α binds Alu elements. We generated a custom protein binding microarray (PBM3) that contained 200 unique Alu-associated sequences (Figure 1). Since RAR was previously shown to bind DR2-like sequences (AGGTCAxxAGGTCA) in Alu repeats [17] and since we have previously shown that HNF4α, while preferring DR1s, can also bind DR2s [43], we also put on the PBM ~1470 permutations of DR1 and DR2 sequences as well as ~150 random controls and ~2000 additional sequences in the human genome predicted by a support vector machine (SVM) algorithm to bind HNF4α [42]. Each sequence was replicated four times for a total of more than 15,000 spots of DNA.

**Figure 2:**
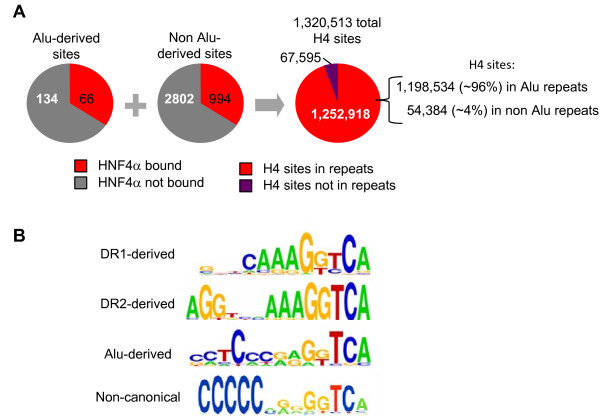
**The vast majority of HNF4α binding sites in the human genome are found in Alu repeats**. **A**. Numerical results from PBM3 described in Fig. 1 and in the text. **B**. Position weight matrices (PWMs) generated with Weblogo [76] of sequences bound by human HNF4α2 in the PBM, categorized by the type of sequence. The DR1-derived PWM was from 994 sequences bound by HNF4α2, the DR2-derived PWM from 50 sequences and the Alu-derived sequences from 66 sequences. The non canonical PWM is from Bolotin et al. [42].

We found that human HNF4α2 bound 66 out of 200 Alu-derived 13-mers in a significant fashion (> 2 SD better than random controls, p-value < 0.045 for the lowest binder) (Figure 2A). It also bound 994 out of 3796 non Alu-derived sequences, although eight of those sequences were subsequently found also to be associated with Alu repeats at a frequency of > 90%. An exact match search of the entire human genome (hg18) with the 1060 sequences that bound HNF4α in the PBM (66 + 994) showed that there are a total of 1,320,513 occurrences of those HNF4α binding sites in the genome and that the vast majority (94.9%, 1,252,918) are in repetitive elements, of which most (95.7%, 1,198,534) are in Alu repeats (Figure 2A). This number is much greater than that previously found for RAR binding sites in Alu elements but that is most likely due to the fact that strict DR1 and DR2 consensus sequences were used for the genomic search [17].

The position weight matrices (PWM) of the DR1- and DR2-derived sequences bound by HNF4α were essentially identical and suggested that for HNF4α the core of CAAAG is more relevant than the AGGTCA half sites (Figure 2B). Interestingly, the PWM of the Alu-derived 13-mers bound by HNF4α did not contain a prominent CAAAG core but did contain an identifiable AGGTCA half site on the right hand side; the left hand portion was primarily C-rich. Overall, the Alu-derived PWM strongly resembled the non canonical HNF4α PWM we identified in our previous PBM study [42], although the association of the non canonical motif with Alu elements was not investigated. A partial list of DNA sequences significantly bound by HNF4α in the PBM and their estimated frequencies in Alu repeats and the human genome (hg18) is given in Table 1 (see additional file 2**: Table S1 **for the complete list of HNF4α-bound motifs associated with Alu repeats).

**Table 1:** Frequency of Alu-derived sequences bound by HNF4α in Alu repeats and the human genome.

Sequence	PBM score	# in Alus	# in hg18	%
TGACCTCGTGATC	0.62	101229	101259	99.97
TGAACCCGGGAGG	0.76	122111	122158	99.96
TGAACCTGGGAGG	0.73	126196	126368	99.86
TGACCTCATGATC	0.68	41447	41545	99.76
TGAACCCGGGAGA	0.61	5700	5716	99.72
TGGGGTTTCACCG	0.70	13385	13428	99.68
TGAACCCGGAAGG	0.69	4520	4535	99.67
TGACCTTGTGATC	0.64	40637	40786	99.63
TGAACTCGGGAGG	0.82	6783	6810	99.60
GCACTTTGGGAGG	0.84	469851	471728	99.60
TGAATCCGGGAGG	0.72	5765	5789	99.59
ACACTTTGGGAGG	0.69	38828	38993	99.58
GCACTTCGGGAGG	0.87	8683	8721	99.56
TGACCTCGCGATC	0.77	775	779	99.49
TGAACCCGAGAGG	0.62	4133	4156	99.45
GTACTTTGGGAGG	0.63	15294	15386	99.40
TGAACCCGGGGGG	9.02	1319	1328	99.32
TGACCTTGCGATC	0.73	411	414	99.28
TGACCTCGTGATT	0.63	2863	2888	99.13
GCACTCTGGGAGG	0.74	13202	13342	98.95
TGAGCCCGGGAGG	0.71	5450	5517	98.79
TGAGCCTGGGAGG	0.69	19616	19895	98.60
TGACCTCGTGACC	0.67	1070	1088	98.35
TGACCTCGTGACC	0.68	1070	1088	98.35
TGAACGCGGGAGG	0.72	642	653	98.32
TGACCTCAAATGA	0.63	8625	8779	98.25

PBM score is the average relative intensity of quadruplicate spots across four arrays. Score >.612 indicates significant binding.

### HNF4α binds Alu repeats in the promoter region of target genes *in vivo*

**Figure 3:**
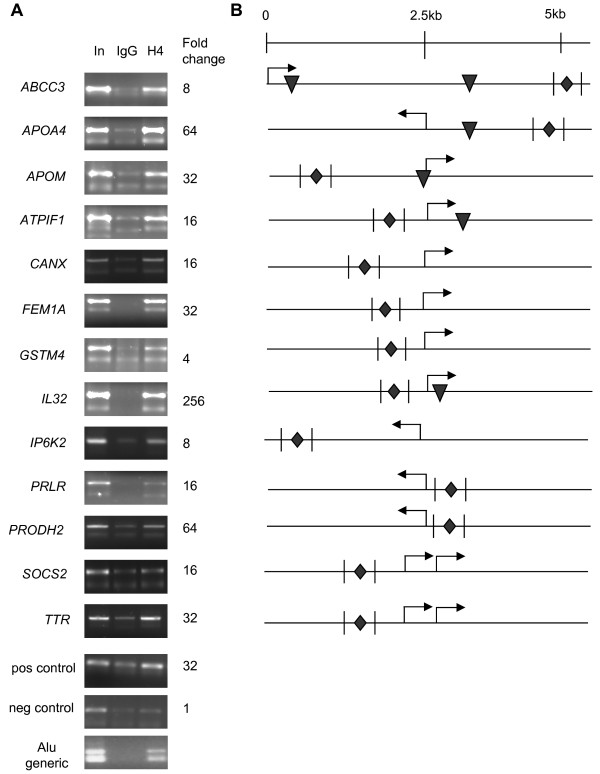
**HNF4α binds Alu elements *in vivo***. **A**. HNF4α chromatin immunoprecipitation (ChIP) of HepG2 cells using 16 sets of PCR primers as indicated. Shown is an ethidium-bromide-stained agarose gel of the qPCR products after ~40 cycles for graphical representation only. In, input control of genomic DNA. IgG, control IP with normal rabbit IgG. H4, IP with HNF4α antibody raised in rabbit. Fold change, ratio of the H4 to IgG signal determined by quantitative real time PCR (qPCR). Pos and neg control, regions of the *CDKN1A *promoter in which HNF4α was shown previously to bind or not, respectively [71]; Alu generic, amplification with generic primers that recognize all Alu elements. Shown are the results from one of two or more independent ChIP experiments performed in duplicate, except for *APOM*, *GSTM4*, *PRLR *and *SOCS2 *which are from one ChIP experiment. The largest fold change values obtained for a given gene are indicated. **B**. Schematic diagram of promoters of HNF4α target genes. Diamonds, position of HNF4α binding sites in Alu elements identified in this study; triangles, other HNF4α binding sites predicted by PBM from Bolotin et al. [42]; vertical lines, position of the PCR primers used in the ChIP; arrows, start sites of transcription. See additional file 2**: Table S2 **for sequences of all the PCR primers.

To investigate HNF4α binding to Alu repeats in the promoters of HNF4α target genes *in vivo*, we performed a ChIP assay for HNF4α in human hepatocellular carcinoma HepG2 cells that express HNF4α and many of its target genes. Several criteria were used for selecting potential Alu sequences for ChIP analysis. First, the Alu element had to contain a probable HNF4α binding site based on the PBM results. Second, the gene containing the Alu element had to be down regulated > 1.4-fold by HNF4α RNAi in HepG2 cells as determined by expression profiling [42]. Third, the Alu repeat had to be within -5 kb to +1 kb of the transcription start site (TSS) of the gene. Fourth, the Alu element had to be amenable to primer design and PCR amplification, non trivial criteria due to the repetitive nature of the sequences. Overall, 47 sets of primers for 35 genes were designed, of which 15 sets gave a specific signal from the input control, indicating appropriate amplification of the Alu sequence. Finally, of those 15 primer sets, 13 genes yielded a significant signal in the HNF4α ChIP assay compared to the corresponding negative control IgG (Figure 3). These results indicate that HNF4α binds the Alu elements in the promoter regions of these target genes *in vivo*.

### HNF4α activates transcription from Alu elements

**Figure 4:**
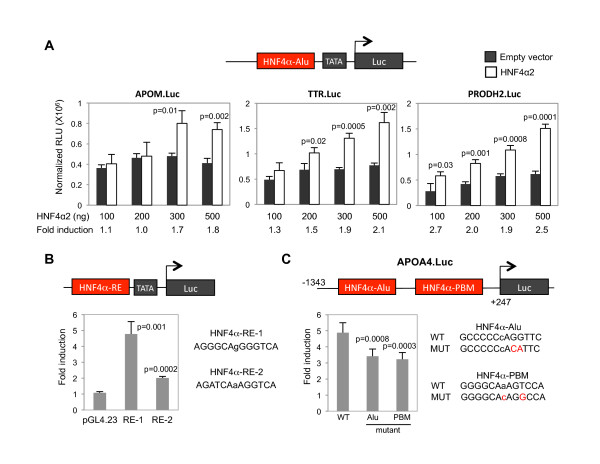
**HNF4α activates transcription from Alu elements**. **A**. Reporter gene assay with the HNF4α-Alu elements from the indicated human gene promoters fused to a minimal core promoter driving luciferase (pGL4.23). Shown is luciferase activity (relative light units, RLU) normalized to β-gal activity (normalized RLU) from HEK 293T cells transiently transfected with 1 μg of reporter and different amounts of either empty vector or human HNF4α2 expression vector (100, 200, 300 and 500 ng). Reporter constructs contain only the HNF4α-Alu element and immediately adjacent sequence; they do not contain any additional known HNF4α binding sites. Data are the mean normalized RLU of triplicate samples from one representative experiment from two or more that were performed. *P*-values of the HNF4α2 signal compared to the empty vector are indicated. Fold induction by HNF4α2 compared to the empty vector is indicated. **B**. As in (A) but with two different reporter constructs containing classical HNF4α response elements (RE-1 and RE-2). Shown is fold induction by 500 ng HNF4α2 compared to the parent construct (pGL4.23). **C**. As in (A) but of the native human *APOA4 *promoter (-1343 to +247) fused to luciferase (pGL4.10) without (WT) or with mutations (MUT) in either the HNF4α-Alu element (Alu) or a classical HNF4α site identified by a previous PBM analysis (HNF4α-PBM) (see Figure 3). Shown is the fold induction by 500 ng HNF4α2 compared to the empty expression vector from one experiment performed in six replicates. A second independent experiment performed in triplicate gave similar results. (**B**) and (**C**), sequence of the relevant HNF4α binding sites are given with the spacer nucleotide in lower case and mutations in red.

In order to determine whether the binding of HNF4α to the Alu elements observed *in vivo *could drive transcription, we subcloned into a luciferase reporter construct with a minimal core promoter the PCR fragments containing the Alu element with the HNF4α binding site (HNF4α-Alu element). Three of the genes ChIP'd by HNF4α in HepG2 cells were analyzed -- *APOM, TTR *and *PRODH2*. Transient transfection into an HNF4α-responsive cell line (HEK 293T) showed that HNF4α2 significantly transactivates the luciferase constructs in a dose-dependent manner (Figure 4A). While the fold induction was not large (1.5 to 2.7-fold), it was comparable to two reporter constructs containing a single classical HNF4α response element (2.0- and 4.8-fold) (Figure 4B). To determine whether an HNF4α-Alu element could contribute to transcription of a native promoter, we analyzed the *APOA4 *promoter construct that contained both an HNF4α-Alu element as well as a classical HNF4α response element. The wildtype (WT) promoter was transactivated well by HNF4α (4.9-fold) and mutations in the HNF4α binding site in either the Alu element or the classical response element reduced the transactivation (to 3.4- and 3.2-fold, respectively) (Figure 4C). While the effect of the mutation in the HNF4α-Alu site was not large, it was statistically signficant (p < 0.001) and comparable to the mutation in the classical site. Taken together, these results indicate not only that HNF4α binds Alu elements in the promoters of HNF4α target genes *in vivo*, but also that this binding can contribute to the overall transcriptional activity of the gene.

### Frequency of HNF4α sites in Alu and non Alu repeats

In order to determine the prevalence of HNF4α binding sites in Alu elements, a search of all the Alu repeats in the human genome (hg18) was performed with the ~1060 (66 +994) sequences bound by HNF4α in PBM3; the vast majority of hits were obtained with the 66 + 8 Alu-derived sequences. Approximately ~750,000 out of ~1,175,000 Alu repeats in Repeat Masker (~64%) were found to contain at least one DNA sequence to which HNF4α bound in PBM3; there was also a substantial number of Alu repeats (~338,000, ~45%) that contained more than one HNF4α binding site (Table 2). All told there were nearly ~1.2 million HNF4α binding sites in Alu repeats in the human genome.

**Table 2:** Number of Alu repeats with HNF4α binding sites in the human genome (hg 18).

# of H4 sites in Alu	# of Alus	Total # of H4 sites
1	409,466	409,466
2	234,592	469,184
3	94,548	283,644
4	8762	35,048
5	232	1160
6	4	24
8	1	8
Total	747,605	1,198,534

**Figure 5:**
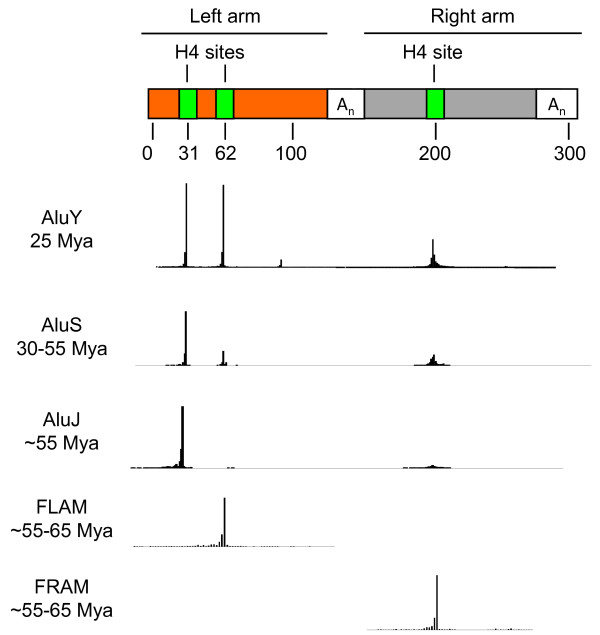
**Distribution of HNF4α binding sites in Alu repeats**. Frequency histogram showing the position of HNF4α binding sites in AluY, AluS, and AluJ families of SINEs as well as the precursors FLAM and FRAM. The approximate age of the repeats is indicated. The slight variation in peaks at positions 31, 62 and 200 are due to small differences in the length of the Alu repeats.

Different families of Alu repeats were found to have different frequencies of HNF4α sites (Table 3) and within a given Alu family there was a range of frequencies (Table 4). There was also a rough negative correlation between the percentage of Alu elements within a given family that contained an HNF4α binding site and the age of the family. The newest Alu family, AluY (~25 Mya), had the greatest percentage of HNF4α sites (~91%); the second newest family, AluS (~30-55 Mya), had the next highest percentage (~75%) and the oldest family, Alu J (~55-65 Mya), had the lowest percentage (~33%) (Table 3) [9]. This correlation held for the precursors to the Alu family as well. FAM (free Alu-like monomer) sequences are Alu precursors that gave rise to FRAM (free right Alu monomer) and FLAM (free left Alu monomer) sequences that eventually joined to create the modern dimeric Alu element [44]. The frequency of the HNF4α binding sites in FAM (0.34%), FRAM (11.84%) and FLAM_C (~33.77%) suggests that the HNF4α sites may have first appeared in Alu-like sequences in the FLAM family. Interestingly, not only does AluJ have a similar frequency of HNF4α sites as FLAM_C, but the HNF4α sites in AluJ are almost exclusively in the left arm at position 31 (Figure 5). In contrast, the newer AluS family has significant secondary sites at positions 62 and 200 while the newest Alu family, AluY, has essentially the same number of HNF4α sites at position 62 as at position 31, although the number of sites at position 200 has remained relatively low. All told, these results suggest that there has been a gain of HNF4α binding sites in Alu elements during the course of evolution. (See **see **additional file 2**: Table S3 **for a complete list of Alu repeats with HNF4α binding sites and their frequency in the human genome.)

**Table 3:** Alu families in human genome (hg18) with HNF4α binding sites.

Alu	# with H4	# in hg18	%
AluY	128,437	140,510	91.41
AluS	507,971	675,017	75.25
AluJ	102,628	307,445	33.38
FRAM	1005	8490	11.84
FLAM_C	7509	22,235	33.77
FLAM_A	39	16,050	0.24
FAM	16	4771	0.34

**Table 4:** Alu subfamilies in human genome (hg18) with HNF4α binding sites.

Alu	# with H4	# in hg18	%
AluYa5	3776	3851	98.05
AluYf5	173	178	97.19
AluY	112,814	118,382	95.30
AluYk4	1720	1831	93.94
AluSg	37,876	40,751	92.94
AluYb9	296	323	91.64
AluYb8	2526	2811	89.86
AluSc	30,426	33,886	89.79
AluSg7	7268	8207	88.56
AluSc8	19,011	21,491	88.46
AluSg4	6493	7347	88.38
AluYc3	492	566	86.93
AluSc5	5709	6766	84.38
AluYf4	1128	1353	83.37
AluSx3	23,542	28,952	81.31
AluSp	39,357	49,055	80.23
AluSx4	4499	5658	79.52
AluSq4	1086	1393	77.96
AluSq2	41,871	54,418	76.94
AluSq	16,472	21,472	76.71
AluYd8	167	221	75.57

The human genome search also revealed ~54,000 occurrences of HNF4α sites in non Alu repeats (Figure 2A). The non Alu repeat families were numerically dominated by repeats referred to as mammalian interspersed repeats (MIRs), LINE2 elements (L2) and Tigger (Table 5). However, while only ~1% of the MIRs and L2s possess an HNF4α binding site, more than ~20% of Tiggers do. In addition, more than 50% of the SVA family of retrotransposons contain at least one HNF4α site, although this is not surprising since these elements contain a portion of an Alu element (s**ee **additional file 2**: Table S4 **for a complete list of frequencies of HNF4α binding sites in non Alu repeats).

**Table 5:** Non Alu repeat families in human genome (hg18) with HNF4α binding sites.

Sorted by number of HNF4α sites				Sorted by prevalence of HNF4α sites			
Transposon	# with H4	# in hg18	%	Transposon	# with H4	# in hg18	%
MIRb	2333	223,605	1.04	SVA_C	215	281	76.51
L2a	2256	169,417	1.33	SVA_B	341	465	73.33
MIR	1586	173,924	0.91	SVA_D	932	1370	68.03
L2c	1455	139,570	1.04	SVA_A	132	259	50.97
Tigger2a	1186	3,316	35.77	(CCGGA)n	1	2	50.00
L2b	1050	96,999	1.08	HSATI	19	42	45.24
FRAM	1005	8,490	11.84	LTR22A	80	187	42.78
SVA_D	932	1,370	68.03	Tigger2a	1186	3316	35.77
L2	898	56,019	1.60	HERV-Fc2-int	1	3	33.33
MIR3	849	90,205	0.94	HERVE-int	80	250	32.00
MIRc	828	102,646	0.81	SVA_F	278	998	27.86
MSTA-int	730	3,104	23.52	Tigger2b_Pri	528	2000	26.40
L1PB1	698	13,202	5.29	PABL_A	126	511	24.66
MLT2A2	646	3,865	16.71	MSTB2-int	22	91	24.18
MER20	608	16,569	3.67	MSTA-int	730	3104	23.52
L1MC1	605	12,979	4.66	MER50	573	2527	22.68
MER50	573	2,527	22.68	LTR14B	78	357	21.85
Tigger2	546	2,735	19.96	MSTB-int	187	866	21.59
Tigger2b_Pri	528	2,000	26.40	MER8	408	1931	21.13
L1M5	482	63,783	0.76	MSTC-int	38	183	20.77
MLT1D	473	20,459	2.31	Tigger2	546	2735	19.96

### Frequency of HNF4α-Alu elements in promoters and DNase hypersensitive sites

Others have shown that the region 5000 bp upstream from the TSS (+1) contains on average 3.63 Alu elements [45]. We analyzed the same promoter region and found that every human gene has on average 2.91 HNF4α-Alu elements, consistent with the overall high proportion of Alu elements with an HNF4α site (Tables 3 and 4). To determine which Alu elements may be accessible, and hence potentially play a role in transcription regulation, we determined the number of HNF4α-Alu elements that reside within DNase hypersensitive regions using datasets from the ENCODE project [46,47]. Genome-wide 46,129 HNF4α-Alu elements (~6.2% of all HNF4α-Alu's) are within DNase hypersensitive regions across mutliple cell lines, with 5458 genes containing one or more HNF4α-Alu/DNase sites in their 5 kb promoter region. ~7000 HNF4α-Alu elements are in DNase hypersensitive regions in HepG2 cells alone (6212 from Rep Track 1 and 8127 from Rep Track 2). While these findings may be an underestimate due to the difficulty of sequencing through repetitive elements, they nonetheless indicate that while the majority of the ~750,000 HNF4α-Alu elements may not be accessible in most cell types, a sizeable portion of HNF4α-Alu elements are in regions of open chromatin and hence may be transcriptionally active.

### Age of Alu repeats in HNF4α target genes

**Figure 6:**
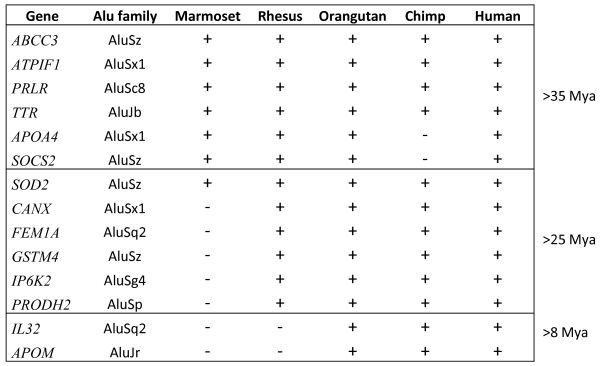
**Gene-specific HNF4α-Alu sequences in primate genomes**. Presence (+) and absence (-) of the HNF4α-Alu element in the indicated HNF4α target genes as determined by ChIP analysis (Figure 3) in all the sequenced primate genomes. Age in millions of years ago (MYA) of the divergence from the primate lineage is given on the right.

**Figure 7:**
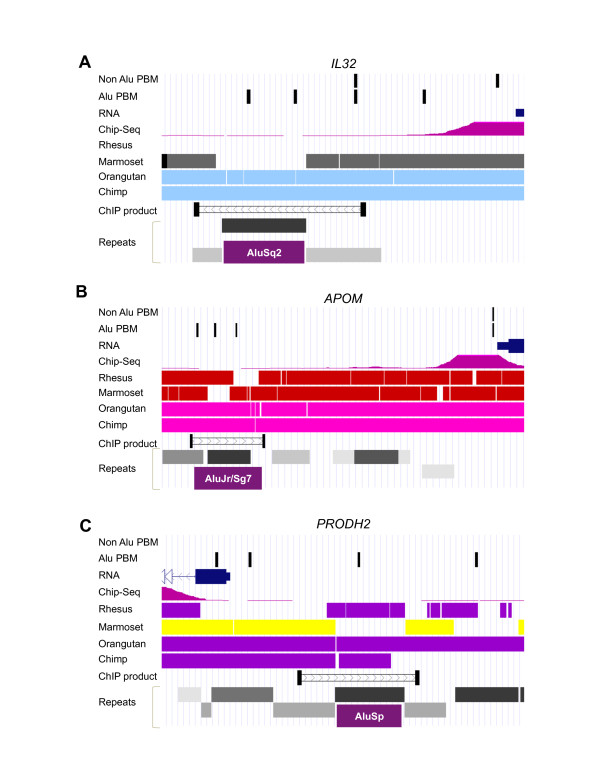
**Alu insertions in the promoters of *IL32*, *APOM *and *PRODH2 *genes**. Screen shots from UCSC Genome Browser of human and other indicated primate genomes in the region of the HNF4α-Alu element ChIP'd by HNF4α in the *IL32 *(**A**), *APOM *(**B**) and *PRODH2 *(**C**) genes. Shown from top to bottom in each figure are non Alu HNF4α binding sites from Bolotin et al. [42] (Non Alu PBM); Alu sites from this study (Alu PBM); mRNA from RefSeq track (RNA); HNF4α ChIP signal in HepG2 from the Custom Track by UC Davis (http://genome.ucsc.edu/ENCODE/) (ChIP-Seq); DNA sequence conservation in four primate genomes (Rhesus, Marmoset, Orangutan, Chimp); PCR product amplified after ChIP in Fig. 3 (ChIP product); repeats from Repeat Masker 3.2.7 with the relevant Alu sequence indicated (Repeats).

In order to estimate the age of the various HNF4α-Alu elements, we determined the presence of the Alu elements bound by HNF4α in the HepG2 ChIP assay in four sequenced primate genomes - marmoset, rhesus, orangutan and chimpanzee. The first mammalian primate originated ~60 million years ago (Mya). The marmoset monkey branched off from the human lineage ~35 Mya, the rhesus monkey ~25 Mya, the orangutan ~8 Mya and the chimpanzee ~5.5 Mya [48]. The results show that all of the HNF4α-Alu elements examined are older than humans (Figure 6), which is not surprising since only ~ 5,000 or 0.5% of Alus are human-specific [9]. Five of the ChIP'd HNF4α-Alu elements (in *ABCC3, ATPIF1, PRLR, TTR, SOD2*) were common among all the primate genomes, and thus fairly ancient (> 35 million years old). An additional two elements (in *APOA4 *and *SOCS2*) also appear to be about 35 million years old but may have been lost after chimps diverged from the primate lineage. In contrast, five of the HNF4α-Alu elements (in *CANX, FEM1A, GSTM4, IP6K2, PRODH2*) appear to be somewhat newer (~25 million years old) due to their presence in all primates except marmoset. The two most recent elements (~8 million years old) appear to be in the *IL32 *and *APOM *genes since they are found only in orangutan, chimp and human. The AluSq2 element in the *IL32 *gene, however, could be older due to the fact that an entire region of the chromosome, including the *IL32 *gene, is missing in rhesus (Figure 7A). In the *APOM *gene, our ChIP results could not distinguish whether the HNF4α site is in AluJr or AluSg7; it is also curious that the AluSg7 element is only partially missing in both rhesus and marmoset (Figure 7B). The AluSp element in the *PRODH2 *promoter, in contrast, appears to have entered the primate lineage after the divergence of the marmoset (35 Mya) but before the divergence of the rhesus monkey (25 Mya), consistent with the reported age of the AluS subfamily (30-55 Mya) (Figure 7C). Assuming that the absence of the HNF4α-Alu elements are not due simply to errors in genome assembly and/or misclassification of Alu elements, these results suggest that HNF4α-Alu elements could play a role in differential regulation of these genes in different primate species.

## Discussion

The functional relevance of repetitive DNA such as Alu repeats in the human genome has been debated ever since they were first discovered several decades ago. In this study, we show that the nuclear receptor HNF4α binds Alu-derived 13-mers *in vitro *as well as Alu elements in the promoters of HNF4α target genes *in vivo*. We show that HNF4α sites in Alu elements can drive gene expression in luciferase assays and that HNF4α binding sites are found in ~64% of all known Alu repeats in the genome (~1.2 million HNF4α sites in ~750,000 Alu elements). Additionally, we found that while HNF4α sites are predominantly found in Alu repeats, they are also found in other repeats such as SVA elements, which contain a portion of Alu repeat [49], and L2, MIR and Tigger families of retrotransposons.

### Functionality of HNF4α-Alu elements

Perhaps the most important question is how many of the HNF4α-Alu elements are functional. Several recent studies suggest that Alu elements may indeed play a role in regulating gene expression: Alu elements are enriched in regions with genes [50], particularly in housekeeping and metabolism genes. However, they are underrepresented in developmental genes [45], suggesting that their presence in those genes may be detrimental. Binding sites for other NRs have also been found in Alu repeats and several of those sites were found to affect transcription [17,19-21]. To determine what types of genes contain HNF4α-Alu elements, we performed a Gene Ontology (GO) analysis of genes enriched with HNF4α-Alu elements (> 8 per 5 kb promoter region) and found RNA processing and transcription regulation genes, as well as macromolecular catabolic processes and complex assembly genes (see additional file 2**: Table S6 **for a full list of significant GO categories and relevant genes). RNA processing is not a category previously associated with classical HNF4α binding sites, but Alu elements have been found to play a direct role in alternative splicing [51].

In a detailed, genome-wide analysis of functional targets of HNF4α and binding sites, we recently found that only 30% of genes down regulated in an HNF4α RNAi experiment contained a potential classical HNF4α binding site [42]. While the other 70% could be indirect targets, it is also possible that some of those genes are regulated by HNF4α-containing Alu elements, consistent with our finding here that on average every gene in the human genome contains ~2.91 HNF4α-Alu elements within 5000 bp upstream of the TSS. On an individual gene basis, we found that even though the HNF4α binding sites in Alu repeats are not high affinity sites compared to the majority of classical HNF4α sites, they are nonetheless capable of driving the expression of a heterologous gene on their own. In the context of the genome, however, the HNF4α-Alu elements are typically present in conjunction with other TFBS in the promoter, including other HNF4α binding sites, suggesting that they may act in more of a modulatory capacity than as the sole drivers of transcription, as we observed on the *APOA4 *promoter. These results are similar to those found for other NRs albeit on different binding sites within the Alu elements [19-21].

The functionality of HNF4α-Alu elements, as with any potential TFBS, will also depend on the state of the local chromatin and the accessibility of the site to HNF4α. While it has been reported that most Alu repeats in the human genome contain CpG dinucleotides that are methylated [52], potentially rendering them nonfunctional, the Alu elements that are hypomethylated tend to be in promoter regions, suggesting that they are accessible [52,53]. Indeed, our analysis showed that there may be as many as ~46,000 HNF4α-Alu elements in DNase hypersensitive regions genome-wide, suggesting that they may be accessible for binding and therefore may affect transcription.

### Alu repeats as a sink for HNF4α protein?

In addition to affecting transcription directly, it is tempting to speculate that the relatively large number of HNF4α-Alu elements, especially in regions of open chromatin, could act as a sink or reservoir for HNF4α protein. We have estimated by semi-quantitative immunoblotting that there may be as many as 450,000 molecules of HNF4α in the nucleus of an adult mouse hepatocyte (unpublished observation); this estimate is consistent with the fact that we originally had to purify HNF4α only ~5,000 to 10,000-fold from adult rat liver nuclei [54]. Assuming that human hepatocytes have similar levels of HNF4α protein and keeping in mind that HNF4α binds DNA only as a dimer [36], this suggests that the presence of ~7000 to 46,000 HNF4α-Alu elements in accessible regions of the genome would not have a significant impact on the availability of ~225,000 HNF4α protein dimers in a normal adult hepatocyte nucleus. However, conditions that significantly alter the accessibility of the ~750,000 HNF4α-Alu elements genome-wide, or the amount of HNF4α protein, could in theory result in a situation in which the stoichiometry of HNF4α-Alu sites to HNF4α protein is indeed relevant. For example, global loss of DNA methylation has been associated with cancer progression and there is at least one report in which certain Alu elements lose methylation during tumor progression [55]. Likewise, a decrease in the amount of functional HNF4α protein, such as that found in heterozygous MODY1 patients [31], activation of signaling pathways [56-61], DNA damage via p53 [62,63], microRNAs [64], diet [35,65,66] and diseases such as colitis and cancer [67,68] could tip the balance between HNF4α protein and potential binding sites, rendering the notion of Alu elements as a sink of HNF4α potentially relevant. The stoichiometry of HNF4α protein to total HNF4α binding sites may also differ in other tissues and developmental time points [69], which could alter the relevance of HNF4α-Alu elements.

## Conclusion

The ~1.2 million HNF4α binding sites in ~750,000 Alu elements in the human genome has the potential to affect the expression of HNF4α target genes. Therefore, it will be important to keep the HNF4α-Alu elements in mind when investigating HNF4α function, especially when using non primates as models for humans and when investigating conditions, such as cancer, where there may be genome-scale alterations in chromatin accessibility. These results join the increasing number of reports of NR and other TF binding sites in Alu or other repeat elements [70] and support the notion that repetitive DNA may be more than just "junk" DNA.

## Methods

### PBM design and analysis

A custom-designed 8x15k Alu PBM (PBM3) containing 8 grids, each of which consisted of ~15,000 spots of DNA, was ordered from Agilent (Figure 1). An *in silico *Alu library of ~200 DNA sequences was made by extracting every unique 13-mer from every Alu element consensus from the RepBase database (http://www.girinst.org/repbase/). The human genome (hg18) was searched with the Alu library and the 100 most frequent sites were included on PBM3. The 13-mer Alu library was further searched with the support vector machine (SVM) model described in Bolotin et al [42]. (The SVM is an algorithm trained on sequences bound by HNF4α in the PBM; it predicts the binding HNF4α binding with correlation R^2 ^= 0.76.) The top 100 scoring potential HNF4α binding sites from the SVM search were included on PBM3 for a total of 200-derived Alu sequences. Another 704 sequences were included from permutations of three adjacent positions in every combination of the DR1 consensus (5'-AGGTCAaAGGTCA-3') and 768 sequences from similar permutations of a DR2 consensus (5'-AGGTCAaaAGGTCA-3'). Additionally, 100 randomly generated 13-mers and 50 randomly generated 14-mers were included as negative controls for the DR1s and DR2s, respectively. Finally, an additional 2,061 unique sequences were generated from an SVM search of all human genes for a total of 3802 unique DNA sequences, each of which was replicated 4 times on the PBM for a total of 15,208 DNA spots. The linker and cap sequences were the same as those described in Bolotin et al. [42]. (See additional file 2**: Table S5 **for a list of all DNA sequences on PBM3 and the corresponding HNF4α binding score.)

Crude nuclear extracts of COS-7 cells transfected with human HNF4α2 or HNF4α8 expression vectors was applied to PBM3 (~400 ng HNF4α protein per grid) and visualized and analyzed as described in Bolotin et al. [42]. The primary antibody was a mouse monoclonal that recognizes the C-terminal region of HNF4α (H1415 from R&D Systems); the secondary was NL-637 anti-Mouse IgG (NL008 from R&D Systems). PBMs were scanned using a GenePix Axon 4000B scanner (Molecular Devices, Sunnyvale, CA) at 543 nm (Cy3) dUTP and 633 nm (Cy5-conjugated secondary antibody). Since there was no significant difference between the HNF4α2 and HNF4α8 isoforms, which differ by ~30 amino acids in the N-terminal region but have identical DNA binding and dimerization/ligand binding domains, the average of the four grids (two with HNF4α2 and two with HNF4α8) were used for the final PBM3 score. The sequences with a score > 0.612 (i.e., 2 SD above the mean of the random controls, p-value < .045) were considered to be HNF4α binders.

### ChIP and RNAi Expression Profiling

HNF4α ChIP from HepG2 cells was performed as described in [71]. Quantitative-PCR (qPCR) following the ChIP was performed using BioRad IQ SYBR Green Supermix. Each 23.5-ul reaction included 12.5 ul of Supermix, 0.25 ul of 100 nmol of each primer, 0.5 ul of template and 10 ul of ddH2O. The qPCR was performed as follows: 95°C for 5 min (hot start), followed by 40 cycles 95°C for 30 sec (melt), 30 sec at the melting temperature (Tm) for annealing and extension, followed by a melt curve. The Tm was determined experimentally for each pair of primers by using a temperature gradient qPCR that was visualized on an ethidium bromide-stained agarose gel to control for product size. All qPCR was performed using BioRad iQ5 and myQ5 thermocyclers. (See additional file 2**: Table S2 **for a complete list of PCR primers giving a positive ChIP signal.) Affymetrix expression profiling data for the HNF4α RNAi knockdown in HepG2 cells were obtained from Bolotin et al. [42].

### Luciferase assay

Human embryonic kidney (HEK 293T) cells were plated (0.25 × 10^6 ^cells) in 12-well plates. After 24 hr the cells were transfected using Lipofectamine 2000 according to the manufacturer's protocol (Invitrogen), with different amounts of empty vector (pcDNA3) or wild type human HNF4α2 in pcDNA3, 1 μg of the luciferase reporter and 200 ng of a CMV.βgal control. Cells were harvested after 24 hr using Triton lysis buffer (1% Triton X-100, 25 mM Gly-Gly pH 7.8, 15 mM MgSO4, 4 mM EGTA, 1 mM DTT). Luciferase and β-gal activity were measured as described earlier [62]. Significant differences in luciferase activity between cells transfected with empty vector or human HNF4α2 were determined by the Student's *t*-test. *APOM*, *PRODH2 *and *TTR *luciferase constructs were created by cloning PCR products of the Alu elements in the respective promoters into pGL4.23 (Promega): the *APOM *construct used SfiI restriction sites and the *PRODH2 *and *TTR *constructs used NheI and KpnI sites. The APOA4.Luc construct was made by cloning a PCR product from the human *APOA4 *promoter (-1343 to +247) into the pGL4.10 vector (Promega) at HindIII and NheI sites. Site-directed mutations were introduced into the HNF4α binding sites in the Alu and PBM elements using the QuikChange kit (Stratagene). Luciferase reporter constructs with classical HNF4α response elements (RE-1 and RE-2) were made by inserting the appropriate synthetic oligonucleotides into pGL4.23. All constructs were sequence verified. (See additional file 2**: Table S2 **for the sequence of the PCR primers and oligonucleotides used in the constructions.)

### Bioinformatic searches

Searches of human genome hg18 downloaded from UCSC Genome Browser (http://genome.ucsc.edu) were conducted using all of the sequences that HNF4α bound in PBM3 using Seqmap [72]. Alu and non Alu repeats with HNF4α sites were identified by comparing the HNF4α genome-wide search results to the repeat coordinates obtained from Repeat Masker Track version 3.2.7 in UCSC Genome Browser. The results were processed using custom Perl scripts and an SQL database. To determine accessibility of HNF4α-Alu sites, we used BEDtools software package [73] to cross reference our list of ~750,000 HNF4α-Alu elements (Table 2) with DNase hypersensitivity tracks in the ENCODE Project in UCSC Genome Bioinformatics, allowing for one nucleotide or more of overlap. We used both the clustered track that contains data from multiple human cell lines (http://genome.ucsc.edu/cgi-bin/hgTrackUi?hgsid=211217271&g=wgEncodeRegDnaseClustered) as well as tracks for two different repetitions of HepG2 cells (http://genome.ucsc.edu/cgi-bin/hgTrackUi?db=hg18&g=wgEncodeUwDnaseSeq). Gene Ontology analysis of genes containing HNF4α-Alu elements was done using DAVID [74]. We used as a cut off eight HNF4α-Alu elements within 5 kb upstream of +1, two SD above the average number of sites (2.91+4.22).

## Authors' contributions

EB designed and carried out the PBM, designed the primers for the ChIP and carried out the PCR, made luciferase constructs, performed all the bioinformatics analysis and drafted the manuscript; KC carried out the luciferase assays and helped with figures; W H-V carried out the ChIP assay; CY made the initial observation of HNF4 sites in Alu elements; JMS made luciferase reporter constructs and mutants; FMS was involved in all aspects of the design of the experiments, analysis of the results and preparation of the manuscript. All authors proof-read the manuscript.

## Supplementary Material

Additional file 1 **Overrepresentation of Alu-related HNF4α binding motif (H4.141) in the human genome**. Frequency profile of 217 HNF4α binding sites identified by gel shift assays and derived from the literature in the human and mouse genomes. Click here for file

Additional file 2 **Supplementary Tables**. Six tables with frequencies of HNF4α binding sites in repetitive DNA, complete PBM and GO results, and primers used in this study. Click here for file
