# Supplementary material for: Nuclear Receptor HNF4α Binding Sequences are Widespread in Alu Repeats
Source: BMC Genomics. 2011 Nov 15;12:560. doi: 10.1186/1471-2164-12-560 (PMC3252374; doi:10.1186/1471-2164-12-560)
Supplement: Additional file 1 — Overrepresentation of Alu-related HNF4α binding motif (H4.141) in the human genome. Frequency profile of 217 HNF4α binding sites identified by gel shift assays and derived from the literature in the human and mouse genomes. [file 1471-2164-12-560-S1.PDF]

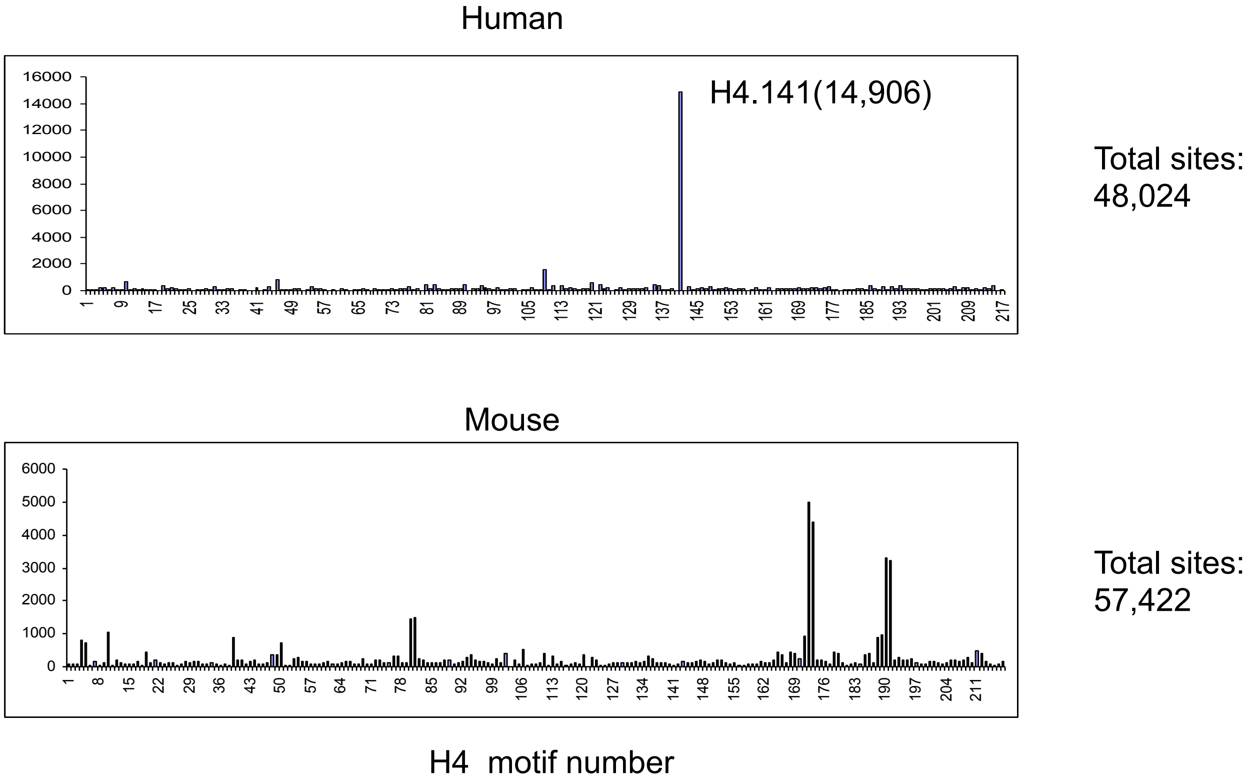

Figure S1. Overrepresentation of Alu-related HNF4α binding motif (H4.141) in the human genome.
Frequency profile of HNF4α gel shift and literature-verified binding sites from Bolotin et al. 2010. Overrepresentation of the H4.141 sequence in the human genome (hg18) vs mouse genomes (mm9) (14,906 vs 58) prompted us to initiate this study.
